# Passive Mechanical Properties of Human Medial Gastrocnemius and Soleus Musculotendinous Unit

**DOI:** 10.1155/2021/8899699

**Published:** 2021-02-09

**Authors:** Ruoli Wang, Shiyang Yan, Marius Schlippe, Olga Tarassova, Gaia Valentina Pennati, Frida Lindberg, Clara Körting, Antea Destro, Luming Yang, Bin Shi, Anton Arndt

**Affiliations:** ^1^KTH MoveAbility Lab, Department of Engineering Mechanics, Royal Institute of Technology, Stockholm, Sweden; ^2^KTH BioMEx Center, Royal Institute of Technology, Stockholm, Sweden; ^3^Department of Children's and Women's Health, Karolinska Institutet, Stockholm, Sweden; ^4^National Engineering Research Center of Clean Technology in Leather Industry, Sichuan University, Chengdu, China; ^5^Department of Physiology, Nutrition and Biomechanics, The Swedish School of Sport and Health Sciences, Stockholm, Sweden; ^6^Karolinska Institutet, Department of Clinical Sciences, Danderyd University Hospital, Division of Rehabilitation Medicine, Stockholm, Sweden; ^7^School of Engineering Sciences in Chemistry, Biotechnology and Health, Royal Institute of Technology, Stockholm, Sweden; ^8^Department of CLINTEC, Karolinska Institutet, Stockholm, Sweden

## Abstract

The *in vivo* characterization of the passive mechanical properties of the human triceps surae musculotendinous unit is important for gaining a deeper understanding of the interactive responses of the tendon and muscle tissues to loading during passive stretching. This study sought to quantify a comprehensive set of passive muscle-tendon properties such as slack length, stiffness, and the stress-strain relationship using a combination of ultrasound imaging and a three-dimensional motion capture system in healthy adults. By measuring tendon length, the cross-section areas of the Achilles tendon subcompartments (i.e., medial gastrocnemius and soleus aspects), and the ankle torque simultaneously, the mechanical properties of each individual compartment can be specifically identified. We found that the medial gastrocnemius (GM) and soleus (SOL) aspects of the Achilles tendon have similar mechanical properties in terms of slack angle (GM: −10.96° ± 3.48°; SOL: −8.50° ± 4.03°), moment arm at 0° of ankle angle (GM: 30.35 ± 6.42 mm; SOL: 31.39 ± 6.42 mm), and stiffness (GM: 23.18 ± 13.46 Nmm^−1^; SOL: 31.57 ± 13.26 Nmm^−1^). However, maximal tendon stress in the GM was significantly less than that in SOL (GM: 2.96 ± 1.50 MPa; SOL: 4.90 ± 1.88 MPa, *p* = 0.024), largely due to the higher passive force observed in the soleus compartment (GM: 99.89 ± 39.50 N; SOL: 174.59 ± 79.54 N, *p* = 0.020). Moreover, the tendon contributed to more than half of the total muscle-tendon unit lengthening during the passive stretch. This unequal passive stress between the medial gastrocnemius and the soleus tendon might contribute to the asymmetrical loading and deformation of the Achilles tendon during motion reported in the literature. Such information is relevant to understanding the Achilles tendon function and loading profile in pathological populations in the future.

## 1. Introduction

The main function of human tendons is to transfer the force generated by muscle contraction to the skeleton, but some tendons, e.g., Achilles tendon, exhibit important elastic and time-dependent characteristics that influence the function of the overall muscle-tendon complex [1]. Therefore, characterization of the mechanical properties of the tendon as well as the entire muscle-tendon unit is of importance to gain a comprehensive understanding of tissues' responses to aging, loading, and gender effects. The Achilles tendon is the largest and strongest tendon in the body. It is formed by separate tendons from gastrocnemius and soleus connected with collagenous linkage [2]. Previous studies have reported that the differences in microstructure of synergistic gastrocnemius and soleus likely lead to intermuscle differences in the muscle-tendon behavior during active movement, and the differences depend on the intensity of the movement [3, 4]. However, it is not clear whether the abovementioned intermuscle differences also existed in purely passive lengthening.

Analyzing the interactive lengthening behavior of the muscle-tendon unit (MTU) allowed us to make an inference about the muscle's tensile behavior, which was challenging to measure *in vivo*. The MTU experiences spring-like properties in a relaxed state [5]. During the movement, both muscle fascicles and tendons contribute to the total changes in the MTU length. Differences in elongation changes in tendon and muscle might influence the overall MTU function (e.g., stretch reflex [6] and the force production [7]). Moreover, the passive tension generated during elongation is physiologically important because it constrains the joint [8] and might be altered due to pathologies. Ultrasonography provides a noninvasive measurement of some human muscle-tendon morphology parameters (e.g., muscle fascicle length and pennation angle). Combining ultrasound imaging with a three-dimensional motion capture system can directly measure tendon length changes during movement (i.e., walking and hopping [9, 10]), which could lead to a better individualized assessment on muscle-tendon mechanics. The mechanical properties of the MTU have been shown to vary between individuals [11] and are affected by a variety of conditions, including aging and pathology [12]. In addition, passive stretching has been commonly used by athletes, old adults, and rehabilitation patients to regain a joint range of motion and increasing flexibility [13]. It is assumed that the reaction torque is caused by stretching the musculotendon units, to which is the summed effect of compressing periarticular tissues and ligaments [14]. Only a few parameters have been reported in the passive condition and mostly for the Achilles tendon (e.g., tendon stiffness [15], Young Modulus [16], and moment arm [17]). To the best knowledge of the authors, no comprehensive *in vivo* data on passive mechanical properties of individual tendon compartment, and muscle-tendon interaction of the synergistic ankle plantarflexors, i.e., medial gastrocnemius and soleus, is available in the literature.

The purpose of this study was twofold: first, to quantify passive mechanical properties of medial gastrocnemius and soleus aspect of the Achilles tendon *in vivo* on a subject-specific basis; second, to investigate the passive extensibility of muscle and tendon. These data will provide a normative reference data for muscle-tendon property alternations in clinical populations.

## 2. Materials and Methods

### 2.1. Participants

Ten healthy subjects volunteered to participate in the study (female/male: 5/5, age: 27.6 ± 2.5 years; weight: 66.7 ± 9.1 kg; height: 171.8 ± 5.8 cm). All participants were physically active on a recreational basis and reported no recent lower limb musculoskeletal injuries. Written consent was given by all participants, and the study was approved by the regional ethics committee, Karolinska Institutet, Stockholm, Sweden. All procedures complied with the Declaration of Helsinki.

### 2.2. Experimental Protocol

**Figure 1:**
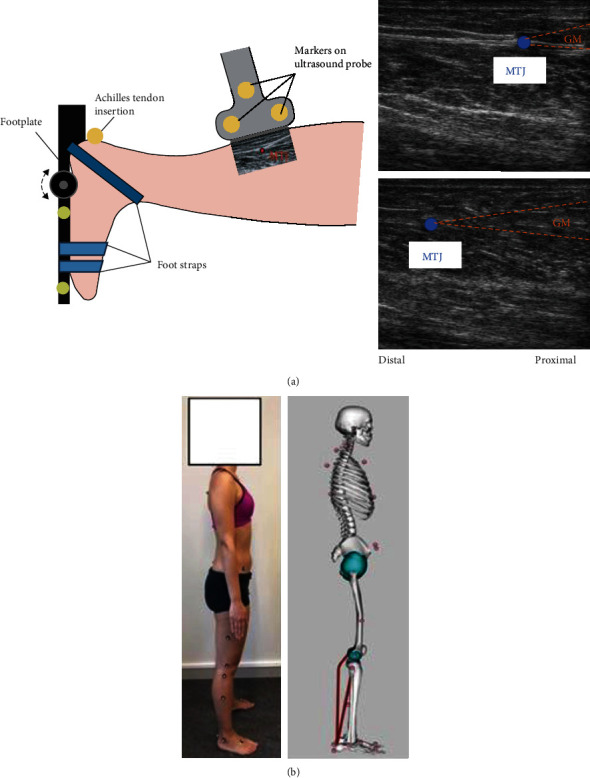
(a) Illustration of the experimental set-up and example ultrasound images during the measurement. The foot is firmly strapped to the footplate of the dynamometer during passive rotation. The ultrasound transducer (US) tracks displacement of the muscle-tendon junction (MTJ) while the motion capture system tracks the location of the reflective markers placed on the US transducer, calcaneus, and the scaffold of the dynamometer. (b) Twenty-seven reflective markers are placed bilaterally on the subject's body landmarks based on a conventional full-body marker set during a static standing reference trial. Marker data is then used to alter the anthropometry of the generic musculoskeletal model to match the subject as closely as possible in OpenSim by the Scale Tool [25]. Musculotendon paths (in red) of medial gastrocnemius and soleus (right limb) are illustrated in a set of straight lines connecting each pair of adjacent points.

The experimental measurement consisted of three parts. First, the subjects were lying in a prone position with their knee flexed at 20° and their foot fixated to a footplate connected to a dynamometer (IsoMed 2000, D&R GmbH, Hemau, Germany). Only the right foot was tested in the convenience of the experimental setup. The ankle joint was carefully aligned with the rotational axis of the footplate with a laser device. In its initial position, the footplate was positioned perpendicularly to the tibia of the subject, which was defined as 0° ankle rotation. Shoulders, hips, legs, and the tested foot were adequately fixated, while paying special attention to securely strapping the foot to the footplate. The test range of motion (ROM) was decided as 30° plantarflexion and 20° dorsiflexion. Prior to testing, the available ROM of each subject was assessed, and no discomfort was discovered within the test ROM. The ankle of the participant was then passively rotated through the test ROM to familiarize the movement, and a static gravitational correction at the neutral position (ankle at 0°) was applied. For the actual measurement, the ankle was rotated at a constant velocity of 5°/s five consecutive times. All participants were instructed to stay relaxed during the passive ankle rotation. Ankle torque generated during the passive rotations and corresponding ankle angle were recorded by the dynamometer at 3 kHz. An ultrasonography system (Mindray M9, Shenzhen, China) with a 38 mm linear transducer (3.5–10 MHz) was used to record muscle-tendon junction (MTJ) excursion of the medial gastrocnemius (MG) and soleus (SOL). The same experienced investigator imaged all the participants. The ultrasound transducer was optimally placed parallel to the tendon in the sagittal plane, and therefore, the ultrasound image plane was therefore aligned with the longitudinal axis of the tendon. To determine the position of the MTJ and the insertion point of the Achilles tendon relative to the global coordinate system, in combination of the ultrasound system, a motion capture system (Qualisys, Gothenburg, Sweden) was utilized. Three reflective markers were placed on the ultrasound transducer, one marker was attached to the skin at the location of Achilles tendon insertion over the calcaneus and two more markers were attached to the IsoMed 2000 footplate appliance (Figure 1(a)) and the marker positions were captured at 200 Hz. Transverse images of the tendon cross-section area (CSA) were also acquired at each muscle's MTJ level during the movement. The surface electromyography (sEMG) signals (Noraxon Inc., AZ, USA) of the MG, SOL, and tibialis anterior muscles were also recorded to exclude muscle activation during the passive movements, and electrodes were placed according to the European recommendations for surface electromyography [18]. Low impedance at the skin-electrode interface was assured by shaving and cleansing the skin with alcohol. The sEMG signals were sampled at a rate of 3 kHz. Second, after the passive ankle rotation, all subjects were asked to perform maximum voluntary isometric contractions (MVC) to establish maximal torque at 0° ankle angle. Other joints were at the same configuration as the passive rotation trial. The MVC was repeated twice with a duration of 5 seconds and with a 30-second rest period. Verbal encouragement from the investigator was provided throughout. The marker positions, sEMG signals, torque, and angle recordings were synchronized analogically and converted to digital data using Spike2 (Cambridge Electronic Design, UK). Raw data of the US were synchronized manually with other recordings and furthermore filtered using a low-pass fourth-order Butterworth filter with a cut-off frequency of 0.75 Hz, 4 Hz, and 14 Hz, respectively. Ultimately, the subjects participated in a static standing reference trial (Figure 1(b)). Twenty-seven reflective markers (9 mm) were placed bilaterally on body landmarks based on a conventional full-body marker set (Vicon Plug-in-Gait).

## 3. Determination of Passive Mechanical Properties of Tendon and MTU

### 3.1. MTJ Displacement and Tendon Length

The location of the MTJ was manually digitized (ImageJ, NIH, Maryland, US) and transformed to the 3D laboratory coordinate system via the use of the three reflective markers mounted on the US probe. Tendon length (*l*_*T*_) of GM and SOL aspects of the Achilles tendon at a specific ankle angle was calculated as the Euclidean distance between the global MTJ position and the insertion point on the calcaneus. The coordinate of the insertion point was then shifted along the longitude axis of the foot by considering the size of the marker [19].

### 3.2. Moment Arm

**Figure 2:**
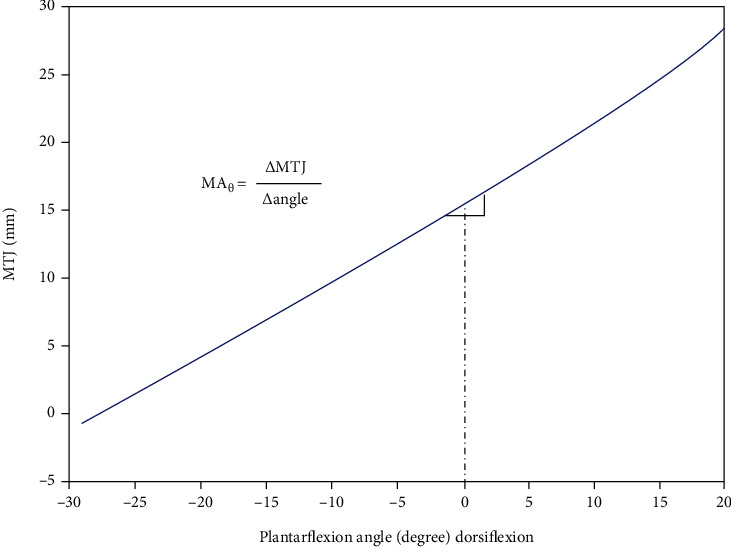
Illustration of the tendon excursion method. The moment arm was calculated as the first derivative of the ratio of the change in muscle-tendon length (*Δ*MTJ) of the medial gastrocnemius and soleus aspect of the Achilles tendon to the changes in ankle angle (*Δ*angle), respectively. For example, moment arm at 0° was calculated as the derivative at 0° over ±1° interval.

The tendon excursion (TE) method was used to estimate the moment arm, which has been well-detailed documented in literature [17, 20]. The TE method is based on the principle of virtual work, which computes the moment arm as the first derivative of the ratio of the change in muscle-tendon length to the changes in the angle of the corresponding joint [17, 21]. A second-order polynomial was first used to fit to the ratio of the change in muscle-tendon length to the change in angle [17]. The tendon elongation was then numerically differentiated with respect to joint angle (over a 2° angle interval) through the ROM (Figure 2).

### 3.3. Tendon Force and Tendon Stress

We assumed that the measured ankle torque was caused by stretching the MTUs of SOL, lateral gastrocnemius (GL), and GM. Other deep plantarflexors were not taken into account. The contribution of the individual MTU (SOL, GL, and GM) to the total ankle torque was assumed to be correlated with the CSA of the individual muscle [22]. The tendon force of the individual MTU was therefore determined as Equations (1) and (2). The CSAs of the SOL, GM, and GL were estimated based on the magnetic resonance images collected earlier for the subjects using a 3T MRI scanner (Siemens Trio, Siemens Medical Solution, Erlangen, Germany) while lying in a supine position with the identical joint configuration. The detail settings for T1-weighted images were described in our previous study [23], and the CSAs were identified at the maximal circumference of the lower limb.

(1) $$\begin{array}{c} F_{T}^{i}=\frac{T_{\text{ankle}}.∙{\text{AR}}_{i}}{{\text{ma}}_{T}^{i}}, \end{array}$$

(2) $$\begin{array}{c} {\text{AR}}_{i}=\frac{{\text{CSA}}_{M}^{i}}{\sum {\text{CSA}}_{M}^{i}}, \end{array}$$

where *i* = SOL, GM, and GL, *F*_*T*_ is the tendon force, *T*_ankle_ is the measured ankle torque, ma_*T*_ is the moment arm, and AR is the ratio of the muscle cross-section area (CSA, Supplementary S2).

Tendon stress was calculated at the MTJ level (*σ*_MTJ_^*i*^) as Equation (3). The transverse US images at the ankle angle of 0° were used to calculate the CSA of the tendon at the MTJ level. The images were outlined manually using ImageJ, and the CSAs were calculated.

(3) $$\begin{array}{c} \sigma_{\text{MTJ}}^{i}=\frac{F_{T}^{i}}{{\text{CSA}}_{\text{MTJ}}^{i}}. \end{array}$$

### 3.4. Slack Length, Force-Strain Relationship, and Stiffness

It was assumed that the resistance to passive stretching of the tendon below its slack length was approximately constant; therefore, the slack length *l*_*s*_ was defined as the tendon length at the slack angle *θ*_*s*_ beyond which a sustained rise in *F*_*T*_ occurred. *θ*_*s*_ was identified independently by two examiners and determined upon the agreement.

Tendon strain was then represented by the engineering strain as

(4) $$\begin{array}{c} e_{T}^{i}=\frac{l_{T}^{i}-l_{s}^{i}}{l_{s}^{i}}. \end{array}$$

A third-order polynomial function was used to represent the force-strain relationship, with the coefficients adjusted using MATLAB (MathWorks, Natick, USA). Tendon stiffness *k* was also defined as the slope of the force-tendon length relationship [24]. To exclude the nonlinear part of the force-strain relationship, the slope was calculated in the region of 20-80% of the maximum passive force *F*_*T*,max_ during the task.

### 3.5. Determination of Extensibility of the MTU

The MTU length (*l*_MTU_) of GM and SOL was estimated by scaling a musculoskeletal model using the static standing reference trial to each subject using OpenSim v.3.3 [25]. The generic musculoskeletal model was previously developed [26] with 14 segments, 23 degree-of-freedom, and 96 musculotendon actuators that characterize the geometry of the bones, the kinematics of lower limb joints, and the paths of muscles (Figure 1(b)). The standard scaling procedures of OpenSim were used [25], where the segment dimensions were determined according to the bone landmarks. The markers of the reference model were then fitted to the captured marker cloud during an upright standing trial. *l*_MTU_ during the passive ankle movement was estimated by placing the scaled model in the same joint configuration as the subject. Muscle length *l*_*M*_ can then be estimated in

(5) $$\begin{array}{c} l_{M}^{i}=l_{\text{MTU}}^{i}-l_{T}^{i}. \end{array}$$

The elongation of each tendon and its corresponding muscle within the tested ROM was defined as

(6) $$\begin{array}{c} ∆l_{T}^{i}=l_{T,\max}^{i}-l_{T,\min}^{i},\\ ∆l_{M}^{i}=l_{M,\max}^{i}-l_{M,\min}^{i}, \end{array}$$

where *l*_*T*,max_^*i*^ and *l*_*T*,min_^*i*^were the maximum and minimum tendon length and *l*_*M*,max_^*i*^ and *l*_*M*,min_^*i*^ were the maximal and minimum muscle length. Therefore, the relative contribution of each tendon and muscle elongation to the whole MTU elongation within the tested ROM can be further determined.

## 4. Data Analysis

In the following, plantarflexion of the foot will be expressed in negative angles and dorsiflexion of the foot will be expressed in positive (+) angles. The fitness between the experimental force-strain relationship and the estimated third-order polynomial function was computed using *R*^2^ (squared correlation coefficient). The Mann-Whitney *U* test was conducted to compare muscle-tendon properties for SOL and GM aspects of the Achilles tendon. A significance level of 0.05 was used for comparison. All statistical analyses were performed using SPSS V25 (IBM SPSS Statistics, Chicago, Illinois).

## 5. Results

### 5.1. Mechanical Properties of Tendons

**Figure 3:**
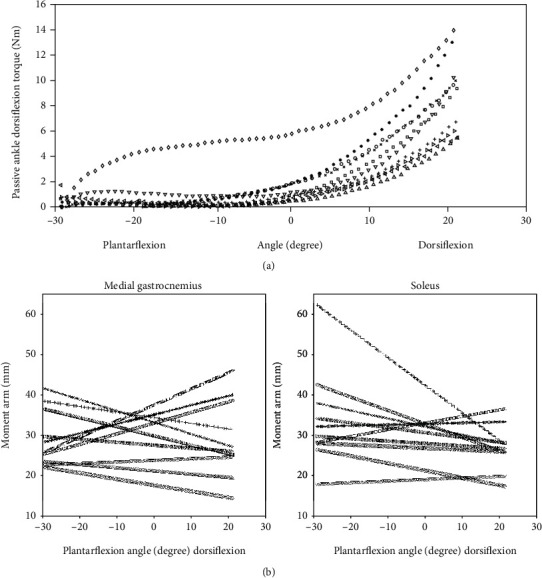
(a) Measured passive ankle torque-ankle angle individual curves of all participants. (b) Ankle moment arms of medial gastrocnemius and soleus aspects of the Achilles tendon in all participants were computed based on the tendon excursion method (TE).

**Figure 4:**
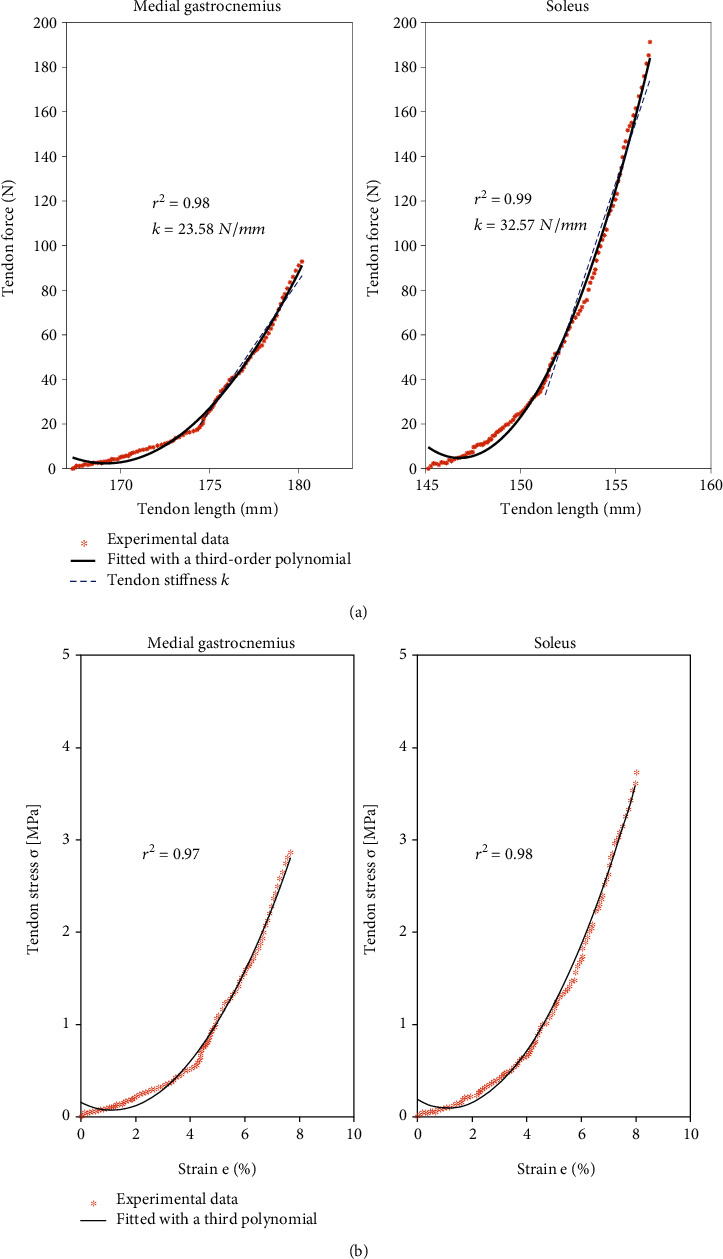
(a) *In vivo* estimated tendon force-length relationship of medial gastrocnemius and soleus aspects of the Achilles tendon for one typical participant. Experimental data (dotted line) was fitted using a third-polynomial function and illustrated in a solid black line. The slope of the fitted tendon force-length curve between 20% and 80% of maximal tendon force was defined as the tendon stiffness *k* (dashed line). (b) *In vivo* estimated tendon stress-strain relationship of medial gastrocnemius and soleus aspect of the Achilles tendon for one typical participant. Experimental data (dotted line) was fitted using a third-polynomial function (solid line).

No active sEMG signals were observed during the measurement in either GM, SOL, or TA (Supplementary S2).  Figure 3(a) shows ankle torque-angle individual curves of all participants. All participants except one had a very similar shape with an initial flat region followed by a nonlinear increase of joint torque. The moment arm of the GM and SOL varied almost linearly but nonuniform among participants from ankle plantarflexion to dorsiflexion position (Figures 3(b) and 3(c)). The mean absolute differences of the moment arm at maximal ankle dorsi- and plantarflexion angle were 9.01 ± 5.06 mm in the GM and 8.30 ± 4.80 mm in the SOL. Figures 4(a) and 4(b) represent the third-order polynomial fitting curve of the tendon force versus tendon length variation, and tendon stress versus tendon strain variation in an example participant illustrated a good fitting with the experimental data. Consistent findings were observed among all participants. The mean determination of the correlation coefficient (*R*^2^) was 0.97 ± 0.02 for GM and 0.97 ± 0.03 for SOL, respectively.

Table 1 summarizes the measured and calculated mechanical or anatomical variables of the GM and SOL aspects of the Achilles tendon in the passive condition. As expected, only a few variables were found to be significantly different between GM and SOL aspects of the Achilles tendon. The mean tendon slack length was found at a slight ankle plantarflexion position. At 20° ankle dorsiflexion, the maximal tendon strain reaches 6.96% and 8.13% in the GM and SOL, respectively. The maximal tendon force of the SOL was found to be significantly higher than GM due to a larger CSA of the SOL. The cross-section area ratio (AR) values were 0.30 ± 0.02 for the GM and 0.57 ± 0.04 for the SOL (Supplementary S2). Thus, maximal tendon stress at the MTJ level of SOL was found to be significantly higher than GM (GM: 2.96 ± 1.50 MPa and SOL: 4.90 ± 1.88 MPa, *p* = 0.024). Moreover, the slack length of the GM tendon was significantly longer than SOL (*p* < 0.01).

### 5.2. Passive Extensibility of Muscle and Tendon

**Figure 5:**
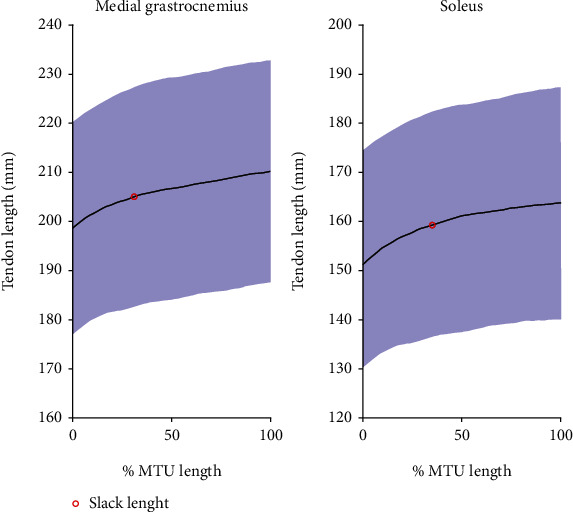
Tendon lengths of medial gastrocnemius and soleus aspects of the Achilles tendon are plotted as the percentage of the muscle-tendon unit (MTU) elongation during the tested range of motion. Tendon slack length is illustrated in a red circle. Solid black lines represent mean values, and blue shades represent the mean ±  1 S.D. values.

**Figure 6:**
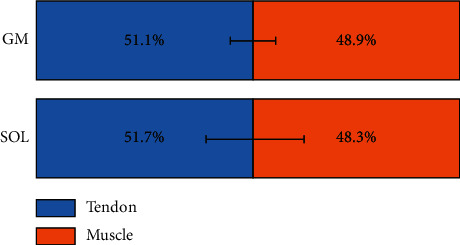
The overall tendon (blue) and muscle (orange) elongation of the medial gastrocnemius (GM) and soleus (SOL) are plotted as a percentage of the muscle-tendon unit elongation during the tested range of motion.

At a very short muscle-tendon length, increases in MTU length were accompanied by a quick increase in tendon length in both the GM and SOL aspects of the Achilles tendon (Figure 5), which also indicated that the tendons were slack at those muscle-tendon lengths. Further lengthening of the MTU resulted in a slower increase in tendon length. The mean tendon slack length occurred at 30.5% ± 13.1% of the physiological range of GM MTU length and 37.3% ± 8.0% in SOL MTU length, respectively. The overall contribution of muscle and tendon elongation to the total MTU elongation was similar in GM and SOL (Figure 6), where the tendon contributed more than half the total MTU lengthening within the tested ROM.

## 6. Discussion

The current study presented the *in vivo* passive mechanical properties of the individual muscle-tendon unit of medial gastrocnemius and soleus in healthy persons using ultrasound imaging combining with a three-dimensional motion capture system. To our knowledge, this is the first study to describe a comprehensive set of *in vivo* mechanical parameters of individual subcompartment of the Achilles tendon. We found that the GM and SOL aspects of the Achilles tendon have similar mechanical properties such as slack angle, moment arm, and stiffness. However, the maximal passive tendon force and stress at the MTJ of GM were significantly lower than in SOL. Regarding individual MTUs, the tendon contributed to more than half of the total MTU lengthening.

Passive tension generated in the relaxed MTU during lengthening is physiologically important, because it constraints joint movement for human locomotion. In many movement disorders, mechanical properties of muscle, tendon, or both altered, which prevents the joint motion necessary for normal motor function. For instance, shortened and stiffer muscle might compensate for the longer and more compliant Achilles tendon in the spastic population [27]. However, the mechanical properties of passive MTU have received much less attention in literature compared to those properties during contraction. Based on our tested ankle ROM and knee configuration, the maximal passive ankle torque was 9.22 ± 3.19 Nm, which agreed with a previous report [5], but smaller than the measurement in a fully extended knee position [16]. The mean elongation of MTU was estimated to be 36.46 mm by scaling a musculoskeletal model using reflective markers, while the mean tendon elongation was 19.13 ± 2.91 mm in GM and 19.21 ± 3.76 mm in SOL. Earlier human *in vivo* studies reported that tendons contributed between half and three-quarters of the total compliance of human gastrocnemius and tibialis anterior MTUs [28]. Tendon contributed to a large part of the total elongation because the tendon is about 10 times as long as the muscle fascicles [8]. Similar findings were also observed in our study, where the tendon contributed to more than half of total MTU lengthening indicating a softer tendon than muscle.

Computational modeling and simulation of the human musculoskeletal system show great promise for improving the diagnosis and treatment of movement disorders [29]. Among others, mechanical properties of the tendon (e.g., slack length and stress-strain relationships) are one of the important parameters implemented into the Hill-type muscle model and to predict passive tension. The accuracy of using generic values or gait-marker-scaled generic musculoskeletal models to obtain muscle-tendon properties has been questioned [30, 31], because muscle-tendon properties do not always scale linearly with bone length [32]. The reliability of simulations is sensitive to these model parameters, which vary considerably between individuals and are challenging to estimate noninvasively *in vivo*. Compared to the active muscle, only a few studies have assessed a relaxed MTU, which can provide important insights into the therapeutic approach for improving joint function in pathological populations.

Tendinous tissues can be considered as a rope, where the slackness as well as the elongation of the rope influences the muscle fiber length and overall joint movability. In past decades, the ultrasonography-based method was considered a golden standard to assess tendon structural and mechanical properties noninvasively [33]. In a combination of tracking tendon displacement using ultrasonography and joint torque measurement using a dynamometer, it was possible to estimate the tension of individual MTU, slack length, and the length-tension properties of the tendon. However, due to variant joint configurations and assumptions, incomparable findings were often reported. For instance, most of the published studies chose arbitrary values for tendon CSA and slack length [34–36]. Tendon slack length is a crucial parameter describing the mechanical behavior of the tendon and also a very sensitive parameter in muscle models, e.g., Hill-type muscle model, to predict passive tendon tension. Tendon slack length is also one of the most challenging parameters estimated *in vivo* [37]. It is now well known that the Achilles tendon slack length does not correspond to the tendon length when the ankle joint angle is at 90° [38, 39] and that CSA is not homogeneous along the tendon [40, 41]. Some studies considered that the passive ankle torque was completely generated by the lengthening of the GM MTU. These authors defined that the tendon slack length was at the angle when the net joint torque was zero, which was reported at 23° plantarflexion when the knee was fully extended [42] and in a less plantarflexion angle around 5° when the knee was 60° flexed. By ignoring other muscle-tendon structures (i.e., SOL and GL) contributing to the joint torque, this approach was likely to overestimate the tension in the single MTU. In this study, we estimated the tension of the single MTU by considering the CSA of medial/lateral gastrocnemius and soleus muscle as well as a nonconstant moment arm. We observed that the slack length of the GM and SOL aspects of the Achilles tendon was at about 10° and 9° plantarflexion. More recently, shear wave elastography has emerged as a new methodology to determine tendon slack length by identifying the onset of the rise of the shear elastic modulus. Hug et al. [38] reported that medial gastrocnemius and Achilles tendon slack length occurs at very different ankle angles, and surprisingly, the Achilles tendon was found having a slack length at a large plantarflexion angle of 44°. Although shear wave elastography was an appealing new image technique in the assessment of tendon mechanical properties, methodological uncertainties, such as application in the inhomogeneous tendinous tissues and saturation in stiff material (e.g., human tendon) as well as localized information, mean that this approach requires further investigation.

The Achilles tendon transmits the force from the main plantarflexors (SOL, GL, and GM) through an intricate system of aponeuroses. It has been recently reported that the force-bearing tissues of the Achilles tendon in humans originate from each of the three muscle compartments and can be mechanically separated well into the free tendon and calcaneal bone. But this feature has received limited attention in general, and whether the mechanical properties of the subcompartment of the Achilles tendon differ has not been investigated *in vivo*. Most published data have been based on the measurement of the free Achilles tendon or GM aspect of the Achilles tendon. By tracking the MTJ displacement of both SOL and GM, we can estimate the mechanical properties of SOL and GM aspects of the Achilles tendon separately. As expected, most of the parameters are similar; the observed significantly greater slack length of the GM aspect was due to the more proximal MTJ location anatomically. Interestingly, we found significantly higher stress in the SOL aspect of the Achilles tendon than GM, which was mainly due to the higher passive tendon force according to our force distribution scheme. There was no consensus on how the tendon forces developing during the passive movement should be distributed; however, it is well-accepted that the force-generating capacity of the three triceps surae muscles is proportional to their physiological CSAs. Therefore, we used the CSA ratio of the muscles to distribute the total passive tendon force. The reason to use the CSA instead of the physiological CSA was that the pennation angles of these three muscles were found rather similar when muscles are at rest. Although this scheme should be further evaluated, our measurement-based observation supports the hypothesis by Bojsen-Møller and Magnusson [43] that muscle-tendon area ratio differences between MTUs are a potential candidate for unsymmetrical loading and heterogeneous deformation in the Achilles tendon during movement. These authors stated that the CSAs of the separate Achilles tendon compartments possibly correspond to the contractile abilities of each muscle compartment while the soleus has the largest physiological CSA.

There are several limitations to this study. The origin locations of GM and SOL were not experimentally tracked due to the limitation of the measurement protocol. Therefore, the MTU length was estimated by scaling a musculoskeletal model using reflective markers. Errors might be induced in the scaling process. However, the mean elongation of the MTU was estimated to be 36.46 mm, which was in the range of the reported data [5]. In addition, the measured passive ankle torque was assumed to be distributed as the ratio of the muscle CSA of LG, MG, and SOL, while other smaller deep muscles such as tibialis posterior, flexor hallucis longus, flexor digitorum longus, and intramuscular tendon were not taken into account. Although Distefano [44] reported that the gastrocnemius together with the soleus was chief ankle plantarflexors and the other plantarflexors only produce 7% of the remaining plantarflexor force, we might overestimate the passive tendon force in the MTU of GM and SOL. In addition, the repeatability in the procedure of manually identifying the tendon slack length might need further investigation.

In conclusion, we have presented a comprehensive set of mechanical property parameters of the GM and SOL perspective of the Achilles tendon in healthy adults. These parameters have important implications in musculoskeletal modeling and provide normative reference data for muscle-tendon property alternations in pathological populations. The unequal stress in the GM and SOL tendon might contribute to the asymmetrical loading and deformation of the Achilles tendon during motion. Such information is relevant for understanding the Achilles tendon function and loading profile in the future.

## Figures and Tables

**Table 1:** Characteristic variables associated with mechanical properties of gastrocnemius and soleus aspects of the Achilles tendon in the passive condition. Significant differences between GM and SOL aspects were denoted in bold.

Variables (mean ± S.D.)	GM aspect of AT	SOL aspect of AT
Slack length *l*_*s*_ (mm)	**205.01 (22.19)**	**159.32 (24.85)**
Ankle angle at the slack length *θ*_*s*_ (°)	-10.96 (3.48)	-8.50 (4.03)
Moment arm at *θ*_*s*_ (mm)	29.58 (5.06)	32.46 (7.46)
Moment arm at *θ*_0_ (mm)	30.35 (6.42)	31.39 (6.42)
MTJ CSA at *θ*_0_ (mm^2^)	36.16 (11.78)	45.61 (20.00)
Maximal passive force (N)	**99.89 (39.50)** ^∗^	**174.59 (79.54)**
Maximal passive tendon stress *σ*_MTJ_ (MPa)	**2.96 (1.50)** ^∗∗^	**4.90 (1.88)**
Maximal tendon strain (%)	6.96 (0.79)	8.13 (0.90)
Tendon stiffness (Nmm^−1^)	23.18 (13.46)	31.57 (13.26)
MTU elongation (mm)	37.32 (4.15)	37.33 (4.17)
Tendon elongation (mm)	19.13 (2.91)	19.21 (3.76)
Muscle elongation (mm)	18.19 (2.23)	18.12 (4.50)
Peak ankle torque during MVC at *θ*_0_ (Nm)	100.72 (30.01)	100.72 (30.01)
Peak force during MVC at *θ*_0_ (N)	1089.24 (320.11)	1309.08 (502.69)

^∗^ *p* = 0.020, ^∗∗^*p* = 0.024.

## Data Availability

The data used to support the findings of this study are available from the corresponding author upon request.

## Supplementary Materials

Supplementary Materials Supplementary S1: example raw EMG signal of the medial gastrocnemius (GM), soleus (SOL), and tibialis anterior (TA) during maximal isometric contraction (MVC) and passive ankle rotation. Supplementary S2: example T1-weighted magnetic resonance image (MRI) of the lower limb at the maximal circumstance of the lower limb. The muscle cross-section area of the medial gastrocnemius (MG), lateral gastrocnemius (LG), and soleus (summation of four subcompartments) was estimated using ImageJ. The ratio of the muscle cross-section area AR (see Method, Equation (2)) was computed for each individual (Table S1). LPS: lateral posterior soleus; MPS: medial posterior soleus; LAS: lateral anterior soleus; MAS: medial anterior soleus.
