# Supplementary material for: Passive Mechanical Properties of Human Medial Gastrocnemius and Soleus Musculotendinous Unit
Source: Biomed Res Int. 2021 Feb 9;2021:8899699. doi: 10.1155/2021/8899699 (PMC7889354; doi:10.1155/2021/8899699)
Supplement: Supplementary Materials — Supplementary S1: example raw EMG signal of the medial gastrocnemius (GM), soleus (SOL), and tibialis anterior (TA) during maximal isometric contraction (MVC) and passive ankle rotation. Supplementary S2: example T1-weighted magnetic resonance image (MRI) of the lower limb at the maximal circumstance of the lower limb. The muscle cross-section area of the medial gastrocnemius (MG), lateral gastrocnemius (LG), and soleus (summation of four subcompartments) was estimated using ImageJ. The ratio of the muscle cross-section area AR (see Method, Equation (2)) was computed for each individual (Table S1). LPS: lateral posterior soleus; MPS: medial posterior soleus; LAS: lateral anterior soleus; MAS: medial anterior soleus. [file 8899699.f1.pdf]

Supplementary S1

Example raw EMG signal of medial gastrocnemius (GM), soleus (SOL) and tibialis anterior (TA) during maximal isometric contraction (MVC) and passive ankle rotation.

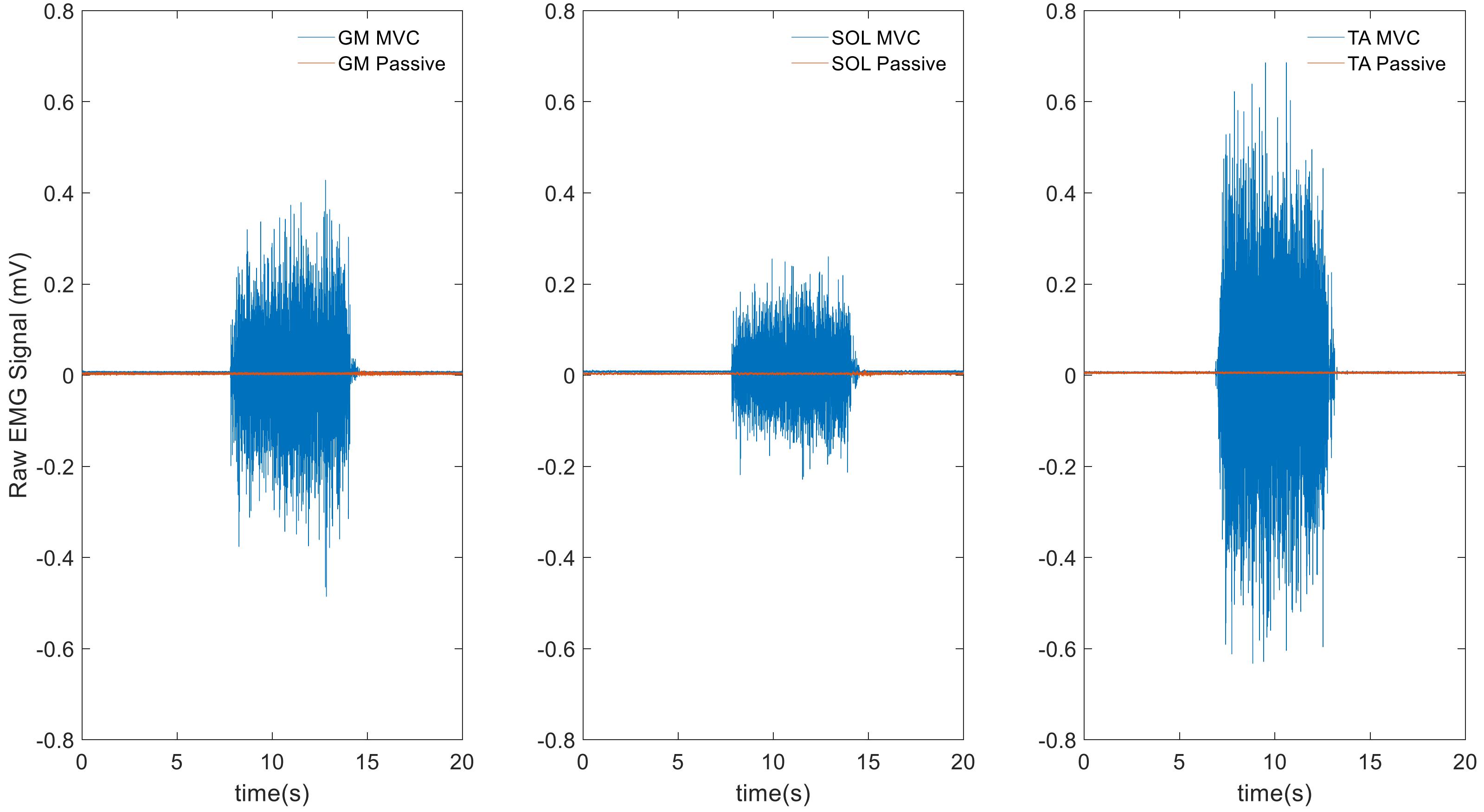

Supplementary S2

Example T1-weighted magnetic resonance image (MRI) of lower limb at the maximal circumstance of the lower limb. Muscle cross-section area of medial gastrocnemius (MG), lateral gastrocnemius (LG) and soleus (summation of four sub-compartments) were estimated using ImageJ. The ratio of the muscle cross-section area AR (see Method Eq.2) was computed for each individual (Table S1). LPS: lateral posterior soleus, MPS: medial posterior soleus, LAS: lateral anterior soleus, MAS: medial anterior soleus.

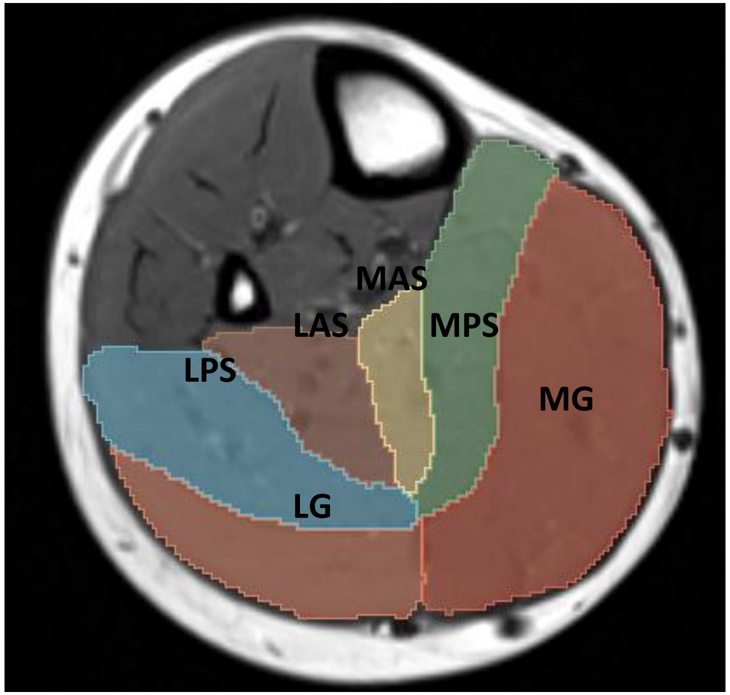

Subject	Cross-section Area Ratio (AR)		
	GM	GL	SOL
1	0.32	0.19	0.49
2	0.28	0.17	0.55
3	0.28	0.11	0.61
4	0.32	0.13	0.55
5	0.28	0.10	0.62
6	0.32	0.06	0.63
7	0.30	0.13	0.57
8	0.32	0.10	0.58
9	0.31	0.13	0.56
10	0.30	0.15	0.55
